# Editorial Note: Short treatment with antalarmin alters adrenal gland receptors in the rat model of endometriosis

**DOI:** 10.1371/journal.pone.0339090

**Published:** 2025-12-18

**Authors:** The PLOS One Editors

The *PLOS One* Editors issue this Editorial Note because this article [1] was identified as one of a series of submissions for which we have concerns about aspects of the peer review process. Readers are advised to interpret the article [1] with caution.

The Editors have no evidence of any author involvement in the peer review process.

## References

[1] Torres-Reverón A, Rana M, Gorabi V, Rivera-Lopez LL, Appleyard CB. Short treatment with antalarmin alters adrenal gland receptors in the rat model of endometriosis. PLoS One. 2020;15(1):e0227456. doi: 10.1371/journal.pone.0227456 31935235 PMC6959558

